# Metabolic activity of visceral adipose tissue is associated with age-related macular degeneration: a pilot ^18^F-FDG PET/CT study

**DOI:** 10.3389/fendo.2023.1322326

**Published:** 2024-01-08

**Authors:** Kwang-Eon Choi, Chanmin Joung, Ki Joo Pahk, Hyunji Kim, Kisoo Pahk

**Affiliations:** ^1^Department of Ophthalmology, Korea University College of Medicine, Seoul, Republic of Korea; ^2^Graduate School of Biomedical Sciences, University of Texas Southwestern Medical Center, Dallas, TX, United States; ^3^Department of Biomedical Engineering, Kyung Hee University, Yongin, Republic of Korea; ^4^Department of Nuclear Medicine, Korea University College of Medicine, Seoul, Republic of Korea

**Keywords:** obesity, age-related macular degeneration, inflammation, visceral adipose tissue, positron-emission tomography

## Abstract

**Background:**

Obesity is known to increase the risk and severity of age-related macular degeneration (AMD). Increased inflamed metabolic activity of visceral adipose tissue (VAT) is considered as a crucial underlying mechanism for the harmful effects of obesity. In this study, we aimed to investigate the inflamed metabolic activity of VAT with ^18^F-fluorodeoxyglucose (FDG) positron emission tomography/computed tomography (PET/CT) and their association with AMD.

**Materials and methods:**

A total of 57 elderly participants (aged ≥ 50 years) who underwent ^18^F-FDG PET/CT for health screening and subsequent fundoscopic exam for complaint of recently impaired vision were enrolled. The metabolic activity of VAT was measured from the maximum standardized uptake value (SUVmax) of VAT. The early AMD participant was defined as the participant with either eye satisfying AMD and without any sign of advanced AMD (neovascular AMD or geographic atrophy). The late AMD participant was defined as the participant with either eye satisfying advanced AMD.

**Results:**

VAT SUVmax was highest in participants with late AMD, intermediate in early AMD, and lowest in non-AMD participants. The levels of systemic inflammation surrogate markers were also highest in late AMD group. Furthermore, VAT SUVmax was positively correlated with systemic inflammation surrogate markers and independently associated with the late AMD.

**Conclusions:**

The metabolic activity of VAT evaluated by ^18^F-FDG PET/CT was associated with the severity of AMD and synchronized with the level of systemic inflammation. Thus, VAT SUVmax could be potentially employed as a surrogate marker of obesity-driven VAT inflammation associated with AMD.

## Introduction

Age-related macular degeneration (AMD) is the leading major cause of visual impairment and blindness worldwide (1). It affects more than 25% of elderly persons aged ≥ 55 years and imposes a substantial economic health burden on the United States ($24.4 billion per year) and the European Union (€89.5 billion per year) (2, 3). Furthermore, until 2050, AMD incidence and prevalence are estimated to increase by 75% and 15%, respectively (4).

Several large population-based cross-sectional studies suggest that obesity contributes to increased AMD incidence and severity in elderly people (5–7). Inflamed visceral adipose tissue (VAT) is one of the key regulators of pathophysiology underlying obesity and AMD (8, 9). Inflamed VAT as a metabolically active endocrine organ secretes proinflammatory cytokines such as interleukin-6 (IL-6), monocyte chemotactic protein-1 (MCP-1), and tumor necrosis factor-alpha (TNF-α), thereby promoting inflammatory cells, mainly macrophages, to infiltrate into VAT, which leads to aggravation of VAT inflammation (10–12). Deteriorated VAT inflammation accelerates increasing systemic circulating proinflammatory cytokines, thereby inducing retinal pigment epithelium injury and photoreceptor death, which eventually lead to AMD (8, 9, 13). Furthermore, normal aging is known to increase systemic inflammation, and VAT is regarded as a key organ associated with the establishment of age-related systemic inflammation (14, 15).

Accumulating studies have shown that ^18^F-fluorodeoxyglucose (FDG) positron emission tomography/computed tomography (PET/CT) is a suitable non-invasive imaging modality for the assessment of metabolic activity of VAT in humans (16–20). Furthermore, in a recent animal study, the metabolic activity of VAT assessed by ^18^F-FDG PET/CT was increased in a mouse model with obesity and could reflect the inflammatory activity of macrophage, which is elevated in inflamed VAT (21). In further clinical studies, we also found that the increased metabolic activity of VAT has been found to be associated with increased tumor aggressiveness and severity of coronary artery disease, for which obesity, especially inflamed VAT, is a well-known risk factor (16–18, 20). Based on these findings, we hypothesized that the metabolic activity of VAT could also be related with the severity of AMD.

In the present study, we aimed to investigate the association between the metabolic activity of VAT as assessed by ^18^F-FDG PET/CT and the severity of AMD in elderly participants who visited a general health screening center.

## Materials and methods

### Study population

**Figure 1:**
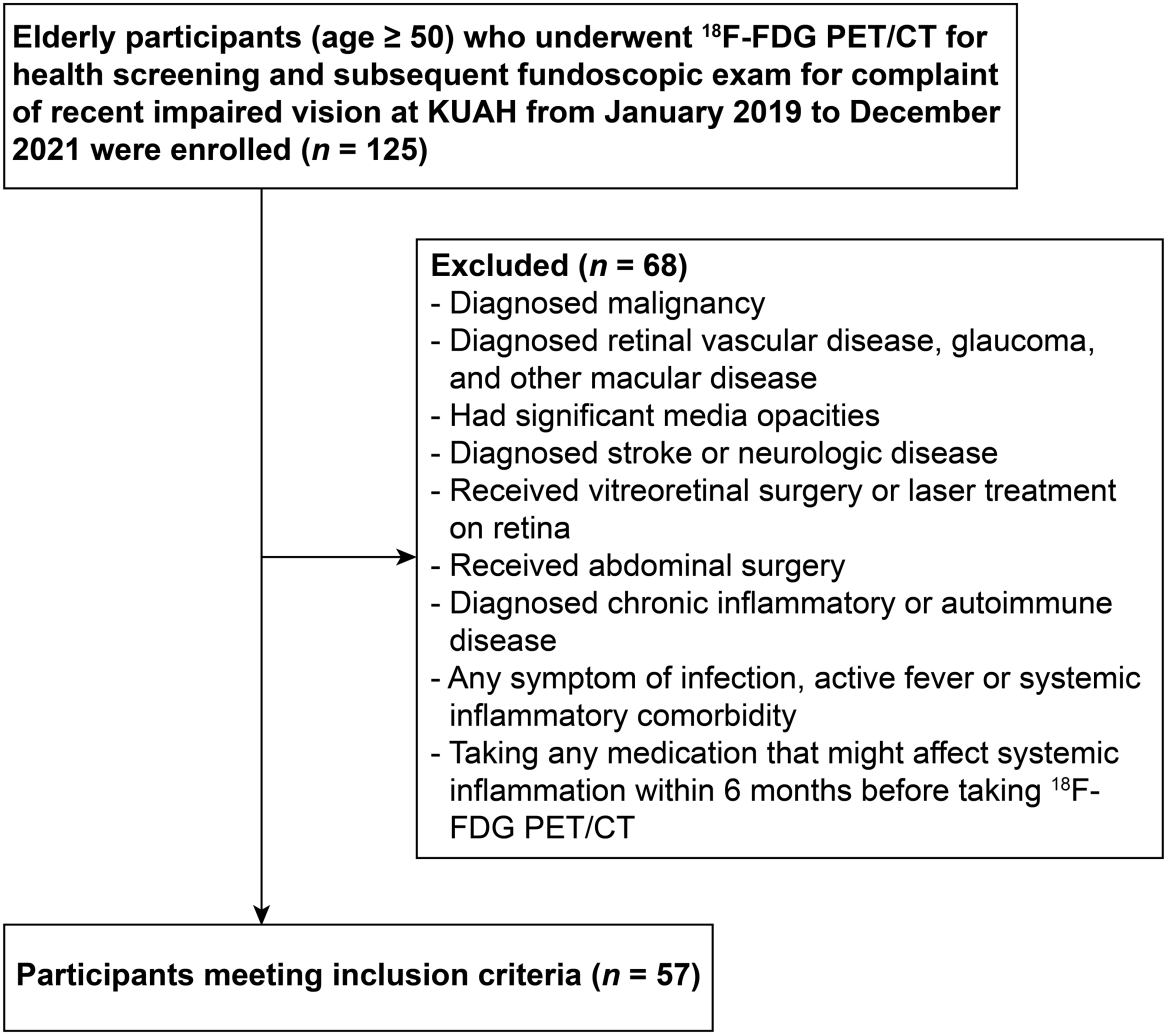
Flowchart showing the enrollment process. ^18^F-FDG PET/CT, ^18^F-fluorodeoxyglucose positron emission tomography/computed tomography; KUAH, Korea University Ansan Hospital.

Elderly participants (age ≥ 50) who underwent ^18^F-FDG PET/CT for health screening and subsequent fundoscopic exam at an ophthalmologic clinic for complaint of recently impaired vision at Korea University Ansan Hospital from January 2019 to December 2021 were enrolled in this study (**Figure 1**). Participants with a malignancy, those who had retinal vascular disease, glaucoma, other macular disease, significant media opacities, diagnosed with stroke or neurologic disease, received vitreoretinal surgery or laser treatment on retina, received abdominal surgery, had any symptom of infection, active fever or systemic inflammatory comorbidity, and those who were taking any medication that might affect the level of systemic inflammation within 6 months before taking ^18^F-FDG PET/CT were excluded. Finally, a total of 57 participants were enrolled in this study. This study conformed to the guidelines of the Declaration of Helsinki and was approved by the Institutional Review Board of Korea University Ansan Hospital (approval no. 2023AS0139). The requirement of informed consent was waived by the Institutional Review Board due to the study’s retrospective design.

### Assessment of AMD

The presence of AMD was determined as follows, according to the Beckman Classification (22): non-AMD: no drusen or small drusen (≤ 63 µm) and no pigmentary abnormalities, early AMD: medium-sized drusen (> 63 and ≤ 125 µm) and no pigmentary abnormalities, intermediate AMD: large-sized drusen (> 125 µm) and/or pigmentary abnormalities, and advanced AMD: neovascular AMD or geographic atrophy.

Although the definition of non-AMD or advanced AMD was consistent across studies, the definition of early and intermediate AMD differed between researchers (23, 24). Thus, to overcome these differences, all non-advanced AMD stages were combined in this study, and the cases were categorized into three clinical stages —non-AMD, early AMD, and late AMD —as previously described (23, 24). Early AMD was defined as participants with either eye satisfying AMD and without any sign of advanced AMD (including early and intermediate AMD). Late AMD was defined as participants with either eye satisfying advanced AMD. All assessments of AMD were performed by a fully experienced retinal specialist (KC).

### Anthropometric and laboratory parameters

Body mass index (BMI) was measured as weight (kg)/height squared (m^2^). All blood samples were gained after 12-h overnight fasting. The levels of total cholesterol, triglycerides, and high-density lipoprotein cholesterol were analyzed using a chemistry analyzer (Hitachi 747, Hitachi, Tokyo, Japan). Low-density lipoprotein cholesterol was measured by the Friedewald formula (25). The levels of high-sensitivity C-reactive protein (hsCRP) were measured by using a chemiluminescence immunoassay (Beckman Coulter, Brea, CA, USA).

### ^18^F-FDG PET/CT protocol

All participants fasted overnight (> 6 h) before undergoing ^18^F-FDG PET/CT. Imaging acquisition was started 1 h after ^18^F-FDG injection at a dose of 5.29 MBq/kg with a dedicated PET/CT scanner (Discovery 710, GE Healthcare, Milwaukee, WI, USA) from the skull vertex to the proximal thigh. The CT scan was performed and immediately followed with a 128-slice PET scan (120 kVp, 60 mA, 2.5 mm thickness) for attenuation correction. All PET images were reconstructed by 3D-ordered subset expectation maximization (two iterations with 16 subsets).

### Image analysis

Image analysis was caried out by two experienced nuclear medicine radiologists (HK and KP) blinded to clinical data using a commercially available workstation (Advantage Workstation version 4.6, GE Healthcare, Milwaukee, WI, USA).

First, both VAT and subcutaneous adipose tissue (SAT) were identified through the predefined Hounsfield units (ranging from −70 to −110) as previously described (16–20). Next, a region of interest (ROI) was located, and a corresponding standardized uptake value (SUV) was acquired as follows:


$$\begin{array}{l} \text{SUV}=\text{Tracer activity}(\text{ROI})(\text{MBq}/\text{mL})/\\ \text{injected dose}(\text{MBq})/\text{total body weight}(\text{g}) \end{array}$$

For the measurement of metabolic activity of VAT, a total of 10 ROIs were located along the intra-abdominal fat boundaries on three consecutive axial slices (between the spine level of L4 and L5) and carefully adjusted to exclude overspill tracer (^18^F-FDG) uptake in the intestine, vessel, and muscle as previously described (16–20). VAT SUVmax was calculated as the averaged maximum SUV from those 10 ROIs. For the assessment of metabolic activity SAT, a total of 10 ROIs were also placed on the buttock and anterior abdominal wall on three consecutive axial slices between L4 and L5 spine levels as previously described (16–20). SAT SUVmax was defined as the averaged maximum SUV from those 10 ROIs.

Heightened ^18^F-FDG uptake of both spleen and bone marrow (BM) is well known to reflect increased myeloid activity, which is closely related with heightened systemic inflammation and thereby useful as a surrogate marker of systemic inflammation (26, 27). For the evaluation of metabolic activity of spleen and BM, ROIs were located on the spleen at whole axial slices and BM of L3 to L5 spine, respectively (26). The averaged SUVmax from those ROIs were defined as spleen SUVmax and BM SUVMax, respectively. In this study, both intra- and interobserver correlation coefficient of the measured SUVs showed excellent reproducibility (coefficient > 0.9).

### Statistical analysis

All data are presented as mean ± standard deviation. Pearson chi-squared (*χ*^2^) test was used for categorical variables. Shapiro–Wilk test was employed to test data normality. One-way analysis of variance (ANOVA) with *post-hoc* Tukey test was used for parametric continuous variables, and Kruskal–Wallis test with *post-hoc* Dunn’s test was used for non-parametric continuous variables. Spearman’s correlation analysis, receiver operating characteristic (ROC) curve analysis, and multiple logistic regression analysis were also performed as statistical methods. MedCalc software version 18.5 (MedCalc Software Ltd., Ostend, Belgium) and SPSS software version 17.0 (SPSS Inc., Chicago, IL, USA) were used for statistical analysis. A *p*-value less than 0.05 was considered statistically significant.

## Results

Of the 57 participants, 23 were in the late AMD group, 19 were in the early AMD group, and 15 were in the non-AMD group. Participants with AMD had a significantly older age than the non-AMD group as expected. The clinical characteristics of all participants are presented in **Table 1**.

**Table 1:** Patients’ characteristics.

No. of patients	Non-AMD	Early AMD	Late AMD	*p*
	15	19	23	
Age (years)	63.8 ± 11.6	72.9 ± 8.9*	76.8 ± 9.6**	0.001
Sex, *n* (%)				0.527
Male	7 (46.7)	11 (57.9)	15 (65.2)	
Female	8 (53.3)	8 (42.1)	8 (34.8)	
BMI (kg/m^2^)	23.4 ± 2.6	23.5 ± 3.9	23.9 ± 4.5	0.909
HTN, *n* (%)				0.791
None	13 (86.7)	15 (78.9)	18 (78.3)	
Yes	2 (13.3)	4 (21.1)	5 (21.7)	
DM, *n* (%)				0.916
None	11 (73.3)	15 (78.9)	18 (78.3)	
Yes	4 (26.7)	4 (21.1)	5 (21.7)	
Dyslipidemia, *n* (%)				0.104
None	8 (53.3)	15 (78.9)	11 (47.8)	
Yes	7 (46.7)	4 (21.1)	12 (52.2)	
Smoking, *n* (%)				0.113
Never	10 (66.7)	14 (73.7)	10 (43.5)	
Ever	5 (33.3)	5 (26.3)	13 (56.5)	
Alcohol, *n* (%)				0.198
Never	12 (80)	13 (68.4)	12 (52.2)	
Ever	3 (20)	6 (31.6)	11 (47.8)	
Cataract surgery, *n* (%)				0.301
Never	10 (66.7)	8 (42.1)	14 (60.9)	
Ever	5 (33.3)	11 (57.9)	9 (39.1)	
Total cholesterol, mg/dL	185.8 ± 52.17	144.5 ± 29.14	165.94 ± 46.21	0.1
Triglycerides, mg/dL	134.3 ± 99.36	85.8 ± 40.2	113.41 ± 48.54	0.12
HDL cholesterol, mg/dL	53.77 ± 19.62	49.13 ± 16.82	49.53 ± 21.1	0.781
LDL cholesterol, mg/dL	92 ± 37.4	78.19 ± 27.45	98.94 ± 41.53	0.378
hsCRP, mg/dL	1.12 ± 2.47	2.28 ± 4.55	3.38 ± 4.78*	0.03
Spleen SUVmax	1.36 ± 0.24	1.86 ± 0.36**	2.24 ± 0.48***^##^	<0.001
BM SUVmax	1.3 ± 0.21	1.78 ± 0.35***	2.1 ± 0.42***^#^	<0.001

AMD, age-related macular degeneration; BMI, body mass index; HTN, hypertension; DM, diabetes mellitus; HDL, high-density lipoprotein; LDL, low-density lipoprotein; hsCRP, high-sensitivity C-reactive protein; SUVmax, maximum standardized uptake value; BM, bone marrow.

*p< 0.05 vs. non-AMD; **p< 0.01 vs. non-AMD; ***p< 0.001 vs. non-AMD; ^#^p< 0.05 vs. early AMD; ^##^p< 0.01 vs. early AMD.

### Metabolic activity of VAT is increased in AMD

**Figure 2:**
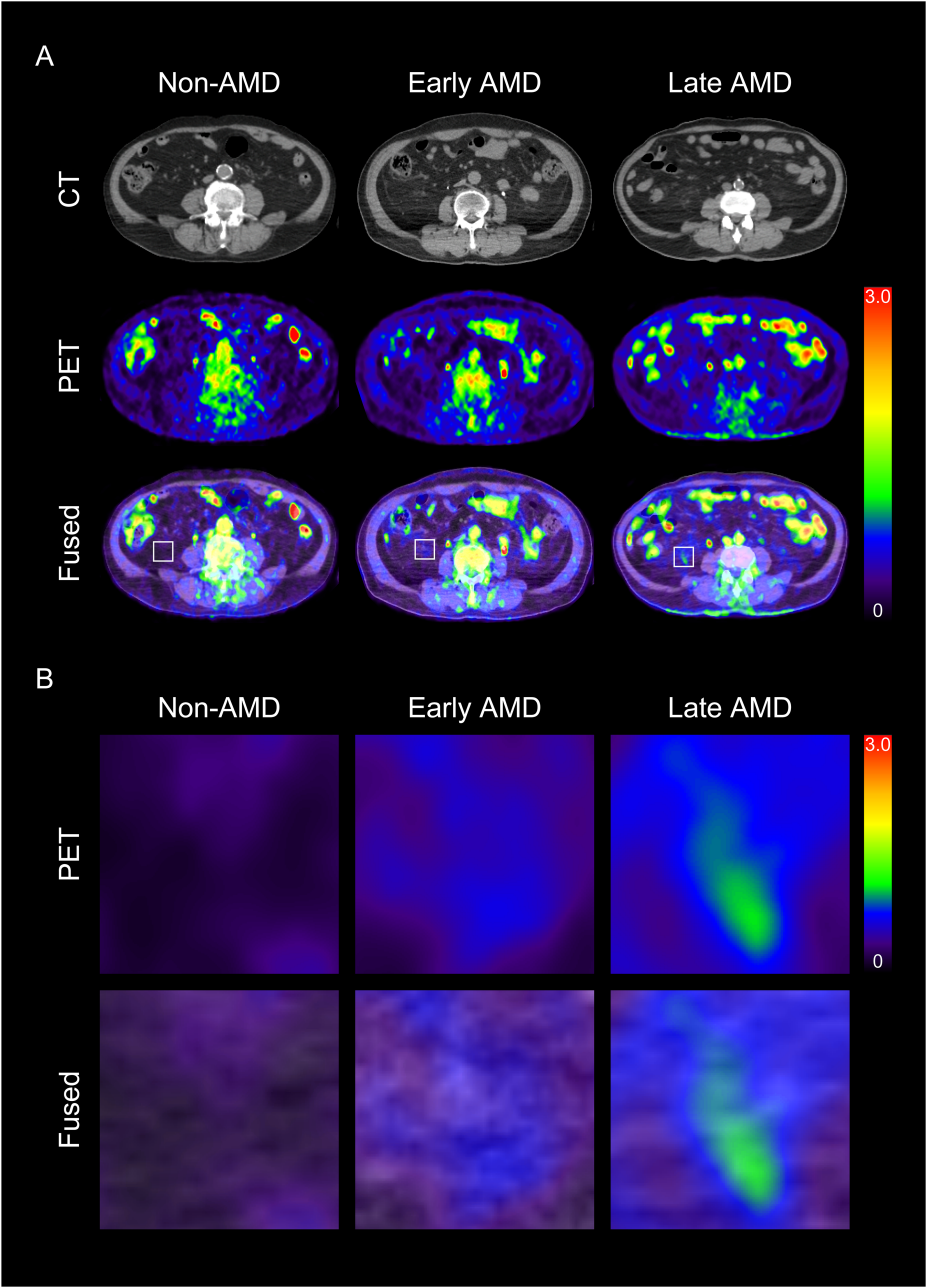
Representative images of visceral adipose tissue (VAT) metabolic activity according to the severity of age-related macular degeneration (AMD) **(A)** and their corresponding magnified images **(B)**. CT, computed tomography; PET, positron emission tomography.

**Figure 3:**
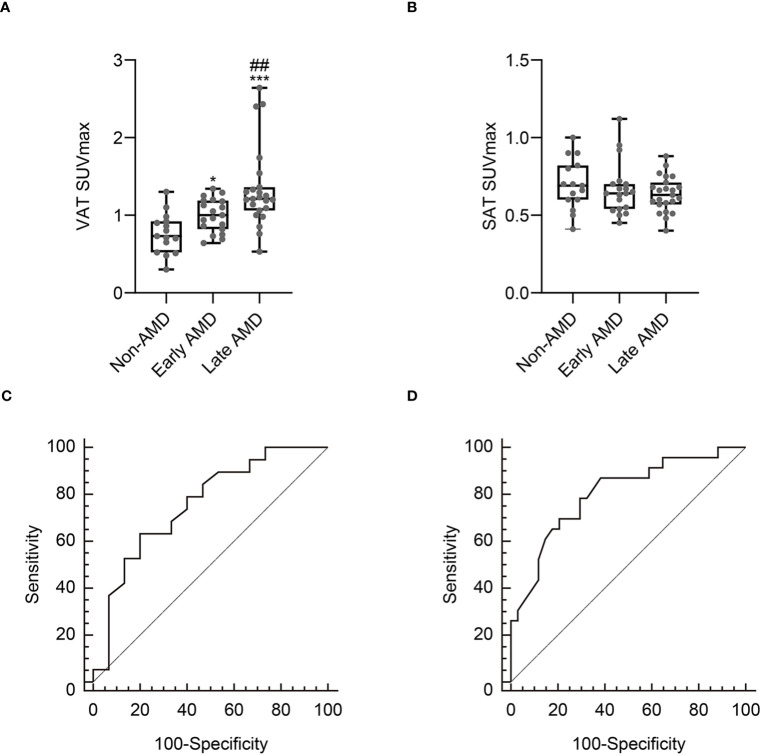
Comparison of VAT SUVmax **(A)** and SAT SUVmax **(B)** according to the severity of age-related macular degeneration (AMD). Receiver operating characteristic curve analysis to identify early AMD **(C)** and late AMD **(D)**. Non-AMD, *n* = 15; early AMD, *n* = 19; late AMD, *n* = 23. SUVmax, standardized uptake value; SAT, subcutaneous adipose tissue. *p< 0.05 vs. non-AMD; ***p< 0.001 vs. non- AMD; ^##^*p*< 0.01 vs. early AMD.

We first investigated whether the metabolic activity of VAT was increased in the AMD group. As shown in **Figures 2** and **3A**, VAT SUVmax was highest in the late AMD group, intermediate in the early AMD group, and lowest in the non-AMD group (1.34 ± 0.52 vs. 1 ± 0.22 vs. 0.76 ± 0.26, *p*< 0.001, respectively). The late AMD group showed a significantly higher VAT SUVmax than the early AMD and non-AMD groups (*p*< 0.01 and *p*< 0.001, respectively). Furthermore, the early AMD group also presented a significantly higher VAT SUVmax than the non- AMD group (*p*< 0.05). In contrast, SAT SUVmax showed no significant difference among the three groups (late AMD: 0.64 ± 0.11, early AMD: 0.67 ± 0.17, and non-AMD: 0.69 ± 0.16; *p* = 0.455; **Figure 3B**). Furthermore, there was no statistically significant difference between the VAT SUVmax and SAT SUVmax of male and female participants in non-AMD (0.71 ± 0.33 vs. 0.81 ± 0.19, *p* = 0.47; 0.64 ± 0.17 vs. 0.74 ± 0.16, *p* = 0.26, respectively), early AMD (0.97 ± 0.24 vs. 1.06 ± 0.18, *p* = 0.398; 0.68 ± 0.22 vs. 0.65 ± 0.03, *p* = 0.7, respectively), and late AMD groups (1.42 ± 0.6 vs. 1.18 ± 0.31, *p* = 0.42; 0.65 ± 0.13 vs. 0.61 ± 0.09, *p* = 0.38, respectively) (**Supplementary Figure 1**).

### Relation between the metabolic activity of VAT and systemic inflammation

Next, we explored whether systemic inflammation was elevated in participants with AMD. As shown in **Table 1**, the levels of systemic inflammation surrogate markers such as hsCRP, spleen SUVmax, and BM SUVmax were significantly increased in participants with AMD than those without AMD. In addition, these levels of systemic inflammation surrogate markers showed a stepwise elevation from the non-AMD group to the early AMD group to the late AMD group. In a further correlation analysis, VAT SUVmax showed a significantly positive correlation with surrogate markers for systemic inflammation, whereas SAT SUVmax showed no significant correlation (**Table 2**).

**Table 2:** Spearman’s correlation analysis.

	VAT SUVmax		SAT SUVmax	
	*r*	*P*	*r*	*p*
Spleen SUVmax	0.789	<0.001^a^	-0.189	0.159
BM SUVmax	0.713	<0.001^a^	-0.166	0.218
hsCRP	0.589	<0.001^a^	-0.054	0.695

VAT, visceral adipose tissue; SAT, subcutaneous adipose tissue; SUVmax, maximum standardized uptake value; BM, bone marrow; hsCRP, high-sensitivity C-reactive protein.

a Statistically significant difference.

### Association between the metabolic activity of VAT and early AMD

According to the ROC curve analysis, as shown in **Figure 3C**, the optimal cut-off VAT SUVmax to identify early AMD was 0.92, with a sensitivity of 63.2% and a specificity of 80% (**Figure 3C**). The area under the curve (AUC) was 0.756 (95% confidence interval: 0.579–0.886; standard error: 0.0867; *p* = 0.003).

Next, we conducted univariate and multivariate logistic regression analyses to investigate the association between VAT SUV max and early AMD using the optimal cut-off value of VAT SUVmax. The univariate analysis revealed that older age and higher VAT SUVmax were significantly associated with early AMD (**Table 3**). In a further multivariate analysis, both older age and higher VAT SUVmax were associated with early AMD, though with marginal significance (*p* = 0.074 and *p* = 0.056, respectively; **Table 3**).

**Table 3:** Univariate and multivariate analysis for early AMD.

Variable	Univariate		Multivariate	
	OR (95% CI)	*p*	OR (95% CI)	*p*
Age (continuous)	1.097 (1.011–1.191)	0.026^a^	1.081 (0.992 – 1.176)	0.074
Sex (male vs. female)	0.636 (0.163–2.487)	0.516		
BMI (continuous)	1.008 (0.822–1.237)	0.938		
HTN (none vs. yes)	1.733 (0.272–11.054)	0.561		
DM (none vs. yes)	0.733 (0.15–3.594)	0.702		
Dyslipidemia (none vs. yes)	0.305 (0.068–1.364)	0.12		
Smoking (never vs. ever)	0.714 (0.162–3.143)	0.656		
Alcohol (never vs. ever)	1.846 (0.376–9.077)	0.451		
VAT SUVmax (≤0.92 vs. >0.92)	6.857 (1.425–33.008)	0.016^a^	5.035 (0.962–26.356)	0.056
SAT SUVmax (continuous)	0.333 (0.005–22.272)	0.608		
Cataract surgery (none vs. yes)	2.75 (0.673–11.239)	0.159		

AMD, age-related macular degeneration; OR, odds ratio; CI, confidence interval; BMI, body mass index; HTN, hypertension; DM, diabetes mellitus; VAT, visceral adipose tissue; SAT, subcutaneous adipose tissue; SUVmax, maximum standardized uptake value.

a Statistically significant difference.

### Metabolic activity of VAT is independently associated with late AMD

To determine the association between the metabolic activity of VAT and late AMD, we first performed a ROC curve analysis to define the optimal cut-off VAT SUVmax to identify late AMD. According to the ROC curve analysis, as shown in **Figure 3D**, the optimal cut-off VAT SUVmax to identify late AMD was 1.13, with a sensitivity of 69.6% and a specificity of 79.4% (**Figure 3D**). The AUC was 0.803 (95% confidence interval: 0.676–0.897; standard error: 0.06; *p* < 0.001).

Next, we performed univariate and multivariate logistic regression analyses to investigate the association between VAT SUVmax and late AMD using the optimal cut-off value of VAT SUVmax. The univariate analysis showed that older age, ever smoking, and higher VAT SUVmax were significantly associated with late AMD (**Table 4**). In a further multivariate analysis, both older age and higher VAT SUVmax were independently associated with late AMD, and VAT SUVmax had the highest odds ratio compared with all other variables (**Table 4**).

**Table 4:** Univariate and multivariate analysis for late AMD.

Variable	Univariate		Multivariate	
	OR (95% CI)	*p*	OR (95% CI)	*p*
Age (continuous)	1.08 (1.017–1.147)	0.012^a^	1.084 (1.007–1.167)	0.033^a^
Sex (male vs. female)	0.6 (0.202–1.786)	0.359		
BMI (continuous)	1.032 (0.897–1.188)	0.658		
HTN (none vs. yes)	1.296 (0.344–4.883)	0.701		
DM (none vs. yes)	0.903 (0.254–3.211)	0.874		
Dyslipidemia (none vs. yes)	2.281 (0.768–6.776)	0.138		
Smoking (never vs. ever)	3.12 (1.032–9.432)	0.044^a^	2.856 (0.671–12.149)	0.155
Alcohol (never vs. ever)	2.546 (0.833–7.788)	0.101		
VAT SUVmax (≤1.13 vs. >1.13)	8.816 (2.612–29.759)	<0.001^a^	5.307 (1.377–20.46)	0.015^a^
SAT SUVmax (continuous)	0.106 (0.002–5.221)	0.259		
Cataract surgery (none vs. yes)	0.723 (0.247–2.118)	0.554		

AMD, age-related macular degeneration; OR, odds ratio; CI, confidence interval; BMI, body mass index; HTN, hypertension; DM, diabetes mellitus; VAT, visceral adipose tissue; SAT, subcutaneous adipose tissue; SUVmax, maximum standardized uptake value.

a Statistically significant difference.

## Discussion

To the best of our knowledge, this is the first human study to investigate the association between the metabolic activity of VAT and the severity of AMD in elderly participants using ^18^F-FDG PET/CT. In the present study, we found that the metabolic activity of VAT defined as VAT SUVmax was highest in participants with late AMD, intermediate in participants with early AMD, and lowest in non-AMD participants. VAT SUVmax presented a significant correlation with systemic inflammation. In addition, it was independently associated with late AMD even after adjusting for all other risk factors.

AMD has been associated with increased serum inflammation markers, including IL-6, IL-1β, CRP, and TNF-α (8, 28, 29). Furthermore, AMD-related genetic risk factors, such as single-nucleotide polymorphisms in both matrix metalloproteinases (MMPs) and complement factor H, are also associated with increased systemic inflammation (30, 31). In both animals and humans, increased VAT inflammation is known to affect the level of systemic inflammation (20, 21). In a recent study, Hata et al. (32) report that macrophages in inflamed VAT could migrate to the distant eye, where they promote an inflammatory cascade that exacerbates the progression of AMD. Therefore, these previous results, in combination with our own, indicate that inflamed VAT is associated with an increased severity of AMD.

In this study, we found that smoking is associated with late AMD, which is consistent with a previous population-based cohort study (33). However, we could not find a significant association between BMI and late AMD. Although BMI is an easily obtainable marker of obesity, it is a crude anthropometric measurement (8, 18). Furthermore, BMI could not fully reflect the inflamed metabolic activity of VAT, a crucial underlying mechanism of harmful consequences of obesity (34, 35). In contrast, ^18^F-FDG PET/CT could reflect the metabolic activity of macrophages, which are a major inflammatory cell type in inflamed VAT (21). Thus, these preceding findings, along with our own, suggest that VAT SUVmax evaluated by ^18^F-FDG PET/CT could be used as a surrogate marker of inflamed metabolic activity of VAT related to the development of AMD besides BMI.

In late AMD, especially neovascular AMD, anti-VEGF treatment is currently recommended as a first-line therapy to prevent vision loss (36). However, it shows limited therapeutic efficacy —only about 30% of patients achieve a substantial vision improvement (37, 38). In addition, approximately 10% of patients exhibited a persistent decline in visual acuity following intravitreal anti-VEFG treatment (39). Thus, there is a significantly unmet treatment need for neovascular AMD. Recently, several population-based multi-cohort studies have reported that high levels of physical activity and exercise can have a protective effect against AMD progression (23, 24, 40). Furthermore, a recent study involving human subjects and animals reports that the beneficial effect of exercise on AMD could be attributed, in part, to its anti-inflammatory effects, particularly in attenuating VAT inflammation (41). Notably, in a previous study, we observed a reduction in VAT SUVmax after 3 months of exercise in women with obesity (19). Therefore, we believe that using VAT SUVmax assessed by ^18^F-FDG PET/CT can be useful to evaluate the therapeutic efficacy of interventions against inflamed VAT in AMD patients.

In this study, we observed that there was no significant difference in VAT SUVmax between male and female subjects in all AMD and non-AMD groups. Hence, gender status seems to have a lesser impact on VAT SUVmax. However, given the varied modulation of VAT biology and metabolism by sex differences (42), a comprehensive study is warranted to further explore the detailed mechanisms underlying the association between VAT inflammation and sex differences.

This study has several limitations. First, this study was retrospective and carried out at a single center, potentially leading to a selection bias. A subsequent extensive prospective study is needed to confirm the results of this investigation. Second, in addition to aging, various factors, including an unfavorable genotype, differences in stress response axes, and lifestyle factors such as physical activity and dietary habits, could influence both VAT SUVmax and the metabolic activity of BM and spleen (12). Third, while ^18^F-FDG PET/CT is a widely recognized imaging modality for assessing the metabolic activity of VAT, we were unable to conduct a histological examination of VAT tissue samples, which could substantiate our observations. Fourth, we were unable to manage every conceivable factor that could influence the absorption of FDG, such as plasma glucose and insulin levels, and the interval after tracer injection until image acquisition. In addition, we were unable to control the temperature before and during the PET scan, which might activate thermogenesis in brown/beige adipocytes, potentially influencing the uptake of ^18^F-FDG. Finally, although there was no statistical difference in age between the early AMD and late AMD groups (**Table 1**), the late AMD group showed a significantly increased VAT SUVmax compared to the early AMD group (**Figure 3A**). Thus, the confounding factor of age could be minimized in the AMD group. However, both AMD groups showed a significantly higher age than the non-AMD group and a significantly higher VAT SUVmax than the non-AMD group (**Figure 3A**). Therefore, as AMD typically occurs in elderly individuals rather than in younger people, it is difficult to completely exclude the confounding effect of age on VAT SUVmax between the AMD and non-AMD groups. Nevertheless, our novel findings mitigated these limitations by employing an exceptional non-invasive imaging technique to investigate the association between the metabolic activity of VAT and the severity of AMD.

Taken together, our findings highlight that the metabolic activity of VAT assessed by ^18^F-FDG PET/CT was associated with the severity of AMD and synchronized with the systemic inflammation which may promote AMD progression. Hence, VAT SUVmax could potentially serve as a surrogate marker of VAT inflammation linked to AMD. Moreover, our findings may offer valuable perspectives on investigating the intricate relationship between inflamed VAT and the severity of AMD.

## Data availability statement

The original contributions presented in the study are included in the article/**Supplementary Material**. Further inquiries can be directed to the corresponding author.

## Ethics statement

The studies involving humans were approved by the Institutional Review Board of Korea University Ansan Hospital (approval no. 2023AS0139). The studies were conducted in accordance with the local legislation and institutional requirements. The ethics committee/institutional review board waived the requirement of written informed consent for participation from the participants or the participants’ legal guardians/next of kin because the requirement of informed consent was waived by the Institutional Review Board due to the study’s retrospective design.

## Author contributions

KC: Conceptualization, Formal analysis, Investigation, Methodology, Writing – original draft. CJ: Formal analysis, Methodology, Visualization, Writing – original draft. KJP: Formal analysis, Writing – original draft. HK: Formal analysis, Writing – original draft. KP: Conceptualization, Formal analysis, Investigation, Methodology, Supervision, Visualization, Writing – original draft, Writing – review & editing.

## Supplementary material

Supplementary Figure 1 Comparison of VAT SUVmax **(A)** and SAT SUVmax **(B)** in male and female participants according to the severity of age-related macular degeneration (AMD). Non-AMD, *n* = 15; early AMD, *n* = 19; late AMD, *n* = 23. SUVmax, standardized uptake value; VAT, visceral adipose tissue; SAT, subcutaneous adipose tissue.
