# Supplementary figures and images for: Metabolic activity of visceral adipose tissue is associated with age-related macular degeneration: a pilot 18F-FDG PET/CT study
Source: Front Endocrinol (Lausanne). 2024 Jan 8;14:1322326. doi: 10.3389/fendo.2023.1322326 (PMC10801050; doi:10.3389/fendo.2023.1322326)

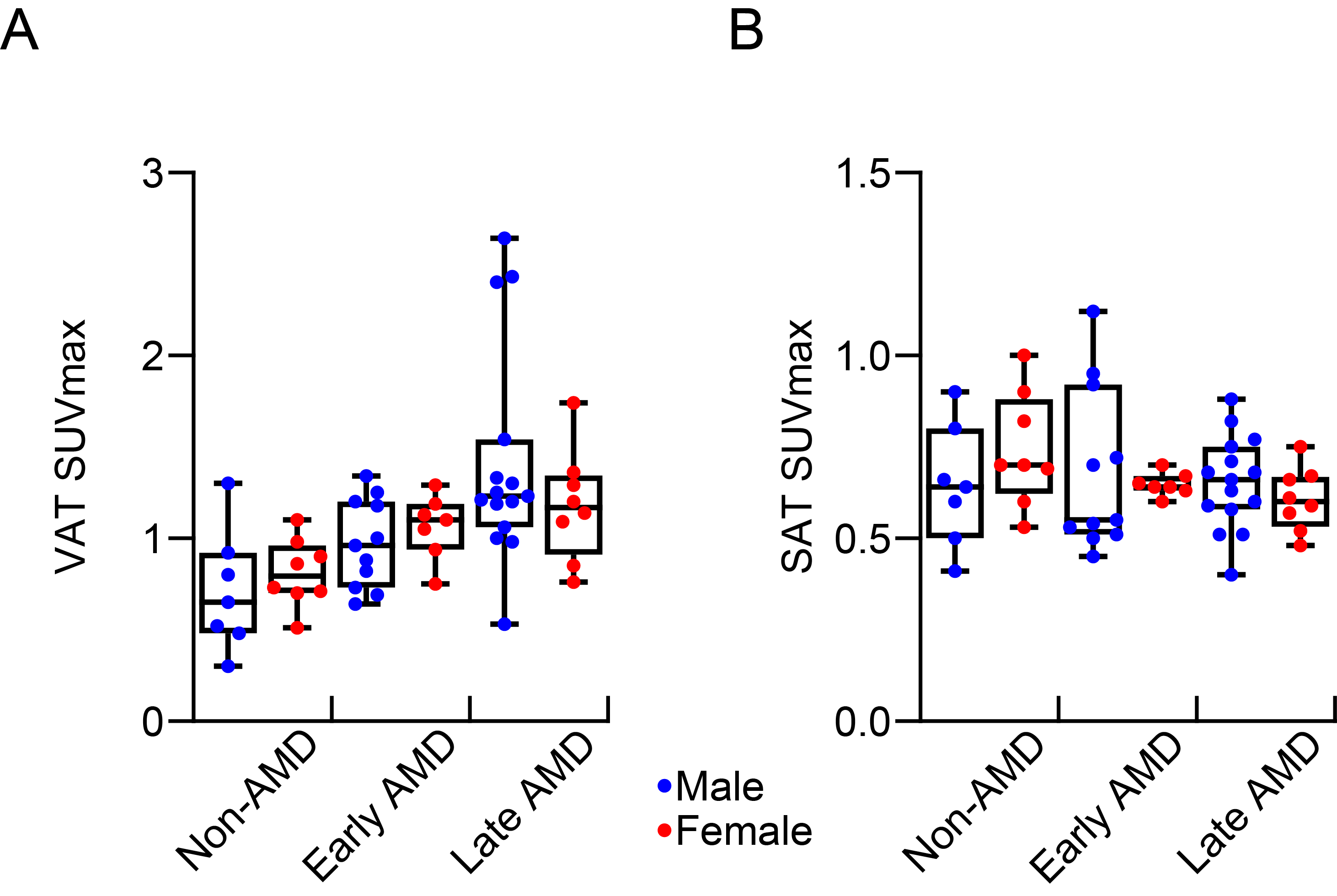

Supplement: Supplementary Figure 1 — Comparison of VAT SUVmax (A) and SAT SUVmax (B) in male and female participants according to the severity of age-related macular degeneration (AMD). Non-AMD, n = 15; early AMD, n = 19; late AMD, n = 23. SUVmax, standardized uptake value; VAT, visceral adipose tissue; SAT, subcutaneous adipose tissue. [file Image_1.tif]
